# Early Hemorrhagic Transformation after Reperfusion Therapy in Patients with Acute Ischemic Stroke: Analysis of Risk Factors and Predictors

**DOI:** 10.3390/brainsci13050840

**Published:** 2023-05-22

**Authors:** Aida Iancu, Florina Buleu, Dana Simona Chita, Adrian Tutelca, Raluca Tudor, Silviu Brad

**Affiliations:** 1 Department of Radiology, “Victor Babes” University of Medicine and Pharmacy, E. Murgu Square No. 2, 300041 Timisoara, Romania; iancuaida22@gmail.com (A.I.); atutelca@gmail.com (A.T.); bradsilviu@yahoo.com (S.B.); 2 County Emergency Clinical Hospital “Pius Brinzeu”, 300732 Timisoara, Romania; tudor.raluca@yahoo.com; 3 Department of Cardiology, “Victor Babes” University of Medicine and Pharmacy, E. Murgu Square No. 2, 300041 Timisoara, Romania; 4 Department of Neurology, Faculty of General Medicine, “Vasile Goldis” Western University of Arad, 310025 Arad, Romania; danaioncu@yahoo.com; 5 Department of Neurology, “Victor Babes” University of Medicine and Pharmacy, E. Murgu Square No. 2, 300041 Timisoara, Romania

**Keywords:** early HT, rtPA thrombolysis, NIHSS, ASPECTS, cerebral computed tomography

## Abstract

***Background***: The standard reperfusion therapy for acute ischemic stroke (AIS) is considered to be thrombolysis, but its application is limited by the high risk of hemorrhagic transformation (HT). This study aimed to analyze risk factors and predictors of early HT after reperfusion therapy (intravenous thrombolysis or mechanical thrombectomy). ***Material and methods***: Patients with acute ischemic stroke who developed HT in the first 24 h after receiving rtPA thrombolysis or performing mechanical thrombectomy were retrospectively reviewed. They were divided into two groups, respectively, the early-HT group and the without-early-HT group based on cranial computed tomography performed at 24 h, regardless of the type of hemorrhagic transformation. ***Results***: A total of 211 consecutive patients were enrolled in this study. Among these patients, 20.37% (*n* = 43; age: median 70.00 years; 51.2% males) had early HT. Multivariate analysis of independent risk factors associated with early HT found that male gender increased the risk by 2.7-fold, the presence of baseline high blood pressure by 2.4-fold, and high glycemic values by 1.2-fold. Higher values of NIHSS at 24 h increased the risk of hemorrhagic transformation by 1.18-fold, while higher values of ASPECTS at 24 h decreased the risk of hemorrhagic transformation by 0.6-fold. ***Conclusions***: In our study, male gender, baseline high blood pressure, and high glycemic values, along with higher values of NIHSS were associated with the increased risk of early HT. Furthermore, the identification of early-HT predictors is critical in patients with AIS for the clinical outcome after reperfusion therapy. Predictive models to be used in the future to select more careful patients with a low risk of early HT need to be developed in order to minimize the impact of HT associated with reperfusion techniques.

## 1. Introduction

Stroke is the second leading cause of death and a major cause of disability in the world [1]. Worldwide, the incidence of strokes is increasing due to the growth of the elderly population, as well as the costs related to this disease [2]. In Europe, the 2015 statistical reports showed that the country with the highest rates of new strokes and deaths due to stroke is Romania [3].

Ischemic stroke accounts for 87% of all strokes, while the remaining 13% is due to hemorrhagic strokes, both of which can lead to life-threatening complications, including death [4]. The growing incidence of strokes globally prioritizes the need for researchers and healthcare providers to understand risk factors, to optimize treatment regimens in order to reduce effects and stroke consequences [5,6].

One of the main adverse complications of AIS (acute ischemic stroke) [7] is hemorrhagic transformation (HT), which can occur spontaneously, after physiologic reperfusion as the natural course of AIS, or after thrombolytic therapy (with rtPA: recombinant tissue plasminogen activator) and/or mechanical reperfusion therapies such as thrombectomy [8]. The incidence of symptomatic HT ranges from 0.6 to 20% [9,10], and in autopsy studies, the incidence is much higher [11]. Hemorrhagic transformation can occur at various timeframes, averaging 6 days (range 1–27) according to a recent study [12]. The term generally used in practice is early hemorrhagic transformation when it occurs at less than 18–24 h, and late hemorrhagic transformation when it develops after more than 18–24 h [13].

In general, strokes can occur for several reasons, including modifiable risk factors (diet, physical inactivity, comorbidities, and environmental conditions), as well as nonmodifiable risk factors (age, genetic predisposition, and gender) [14,15]. Moreover, not only are these risk factors increasing the risk of having a stroke, but studies have shown that they are also associated with severe outcomes or stroke recurrence [16,17].

The incidence of HT after reperfusion is 10 times higher compared to the incidence after spontaneous reperfusion [18] and is increased with time (the later the reperfusion, the higher the HT rate) [19], age [20], stroke severity (assessed with the National Institutes of Health Stroke Scale (NIHSS)) [20], high systolic blood pressure, acute high glucose level, antiplatelet or dual antiplatelet usage before, obesity [21], lower platelet count and by the presence of other comorbidities (atrial fibrillation, hypertension, and diabetes mellitus) [22,23,24]. Therefore, it is critical to investigate how to predict HT in patients with acute ischemic stroke before reperfusion therapy. Furthermore, knowing these modifiable and nonmodifiable risk factors, as well as assessing predictors, is crucial for improving the outcomes of these patients and their quality of life. Thus, the present study aims to (1) analyze which of the major risk factors (conditions/factors present in the patient) and predictors (characteristics or complications that occur during the AIS event) are associated with early HT and (2) assess the incidence of early HT in patients with acute ischemic stroke treated with reperfusion therapy (intravenous thrombolysis or mechanical thrombectomy).

## 2. Material and Methods

### 2.1. Study Population, Inclusion, and Exclusion Criteria

For the present study, we reviewed all electronic and paper medical records covering admission to the emergency department (ED), hospitalization, including administered treatments of consecutive patients aged 18 years or older with AIS who performed thrombolysis (and in whom according to the local protocol [25], intravenous thrombolysis with rtPA was initiated only when the time interval from the first symptoms of stroke to the time of administration is less than 4.5 h) or endovascular treatment through mechanic thrombectomy, in order to analyze the early hemorrhagic transformation of AIS due to reperfusion therapy. All patients from this study were hospitalized at the County Emergency Hospital “Pius Brînzeu” Timisoara, Romania in the period 1 January 2019 and 31 December 2021 and all were part of the National Program of Priority Actions in the Interventional Treatment of Patients with Acute Cerebral Vascular Accident (PA-CVA), founded in 2018 by the Romanian Neurology Society board [6,26].

The study was approved by the Ethics Commission of the County Emergency Hospital “Pius Brînzeu” Timisoara, Romania (approval certificate 385/10 March 2023), and conducted in accordance with the principles of the Declaration of Helsinki. Informed consent was obtained from all subjects involved in the study.

All patients who were admitted to our hospital diagnosed with AIS underwent the necessary investigations, including the tests required to determine the underlying cause of the stroke, according to standardized local protocols and established guidelines [25,26]. AIS management was streamlined and involved the Departments of Neurology, Radiology, and Emergency. Based on the initial and subsequent clinical condition and the results of the CT scan, the neurosurgery team was involved in further interventions, where necessary.

Patients were excluded from this study if they were aged below 18 years and/or had an initial diagnosis of intracerebral hemorrhage and/or brain tumor. Patients who developed HT after more than 24 h or the onset of HT was unclear and/or with incomplete information were also excluded.

### 2.2. Evaluation of Stroke

In all patients, cerebral computed tomography (CT) examination with or without contrast was performed as soon as possible after admission to the Emergency Department. The subtype, severity, and location of the stroke were diagnosed by the radiologist who performed the brain imaging in collaboration with the neurologist who performed the clinical examination.

The diagnosis of AIS was made based on The World Health Organization’s definition of stroke (introduced in 1970 and still used) defined as a “rapidly developing clinical signs of focal (or global) disturbance of cerebral function, lasting more than 24 h or leading to death (unless interrupted by surgery or medication), with no apparent cause other than that of vascular origin” [27,28].

**Figure 1:**
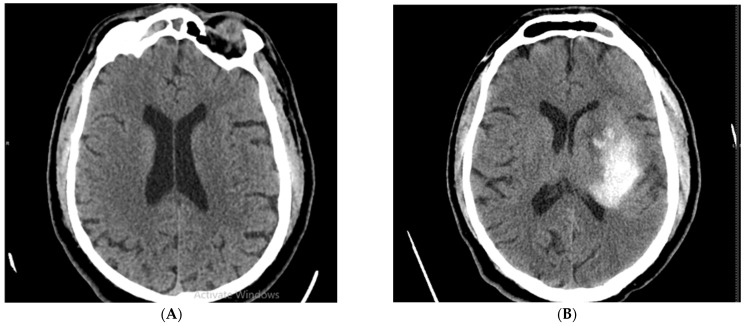
(**A**) Cerebral CT performed at admission in ED and (**B**) Hemorrhagic transformation on Cerebral CT performed at 24 h after performing thrombolytic therapy at the same patient.

Strokes were classified as ischemic strokes and hemorrhagic strokes according to imaging results. The first image below represents a cerebral CT scan performed on admission to the emergency department (ED) on a patient with neurological symptoms eligible for thrombolysis with no established brain lesions detected (Figure 1A). When performing brain CT in the same patient who did not have neurological deterioration, on the cerebral CT performed 24 h after the thrombolytic administration, an intraparenchymal hematoma is detected in the territory of the left middle cerebral artery (Figure 1B).

### 2.3. Evaluation of Hemorrhagic Transformation

Both clinical and radiological criteria were used to classify HT. Symptomatic intracranial hemorrhage (sICH) from asymptomatic intracranial hemorrhage (aICH) was differentiated by clinical criteria. sICH was defined as a worsening of the National Institutes of Health Stroke Scale (NIHSS) attributable to HT by ≥4 points within the first 24 h of stroke onset. The limitation of the clinical classification is the omission of HT when there is no major neurological decline. This may be the case when reperfusion therapy improves NIHSS and compensates for potential worsening caused by HT. The radiological criteria used for HT originated from the European Cooperative Acute Stroke Study (ECASS) and distinguishes two stages: hemorrhagic infarction (HI) and parenchymal hemorrhage (PH) with or without mass effect. Each stage is divided into two subtypes [29].

For this study, records of patients who performed reperfusion therapies were accessed, along with findings from repeated scans for suspected neurological deterioration and hemorrhagic transformation. Patients who developed HT within 24 h were labeled as early HT, and those detected after 24 h were labeled as late HT [30].

Neurological deficit was assessed and categorized using NIHSS at the time of admission, at 1 h, 2 h, and 24 h. NIHSS scores were represented by the following values, respectively 0 = no stroke symptoms, 1–4 = minor stroke, 5–15 = moderate stroke, 16–20 = moderate/severe stroke, and 21–42 = severe stroke [31]. According to the European Cooperative Acute Stroke Study (ECASS) III classification of HT, symptomatic HT is defined by an increase of at least 4 points in the NIHSS [32].

To detect early ischemic changes on CT scans of the brain without contrast, in patients who are suspected of occlusion of the anterior circulation, The Alberta Stroke Program Early CT score (ASPECTS) was calculated and used as a screening tool for eligibility in receiving reperfusion therapy. ASPECTS consists of a 10-point quantitative score. For any evidence of early ischemic change for each of the defined regions, 1 point is subtracted from 10. A score of 0 indicates infarction of all 10 regions. Low ASPECTS (<7) indicate large infarctions associated with increased stroke severity and increased risk of HT [33].

### 2.4. Reperfusion Therapy Protocol

Fibrinolysis was performed in the Emergency Department, in order to avoid further delay of thrombolysis and to be able to quickly transport the patient to the CT scanner in case of clinical deterioration during the reperfusion. After the completion of fibrinolysis, the patient was transported to the Neurology department where the patient was monitored for at least 24 h.

The patients not eligible for endovascular treatment (time elapsed from the onset of symptoms between 4.5 and 6 h for stroke in the anterior cerebral artery territory, respectively 4.5 and 12 h for stroke in the posterior cerebral artery territory or patients with contraindications for intravenous thrombolysis) were transported to the Department of Interventional Radiology, were mechanical endovascular thrombectomy was promptly performed under general anesthesia by a neurointerventional radiologist. The patients were immediately extubated after the procedure was completed and no patients from this study remained on mechanical ventilation. After mechanical endovascular thrombectomy, patients were admitted to our Intensive Care Unit for careful monitoring and treatment at target blood pressure (BP = 135/80 mmHg) and saturation (Spo2 = 94–98%) depending on individual therapeutic needs. After discharge from the ICU, patients were admitted to the stroke unit for further clinical monitoring performed by neurologists.

### 2.5. Stroke Risk Factors

The study data were collected on stroke risk factors: age, gender, smoking (defined as current use of >1 cigarette per day), alcohol consumption, body mass index (BMI) (calculated according to the following formula: BMI = weight (kg)/height^2^ (m^2^), history of hypertension (blood pressure value > 140/90 mmHg at least twice before stroke or already under treatment with antihypertensive drugs), history of diabetes mellitus (glucose level > 126 mg/dL pre-prandial on two examinations, glucose level > 200 mg/dL postprandial or already under treatment with antidiabetics drugs), clotting parameters, hyperlipidemia and the presence of atrial fibrillation [AF]. These data were recorded at the time of admission for all patients.

### 2.6. Statistical Analysis

Continuous variables were presented as mean and standard deviation or median and interquartile range (Q2–Q3), and categorical variables were presented as frequency and percentages. To check the distribution of continuous variables we employed the Shapiro–Wilk test. To compare patient’s characteristics with and without hemorrhagic transformation we employed an unpaired *t*-test or Mann–Whitney U test and Chi-square test. In order to check the independent factors associated with early-hemorrhagic transformation we employed logistic regression (Enter method). Odd ratio and 95% confidence interval were presented. A *p*-value < 0.05 was considered statistically significant. Data analysis was performed using IBM SPSS Statistics version 26.0 (IBM Corp, Armonk, NY, USA).

## 3. Results

### 3.1. Incidence of Hemorrhagic Transformation

**Figure 2:**
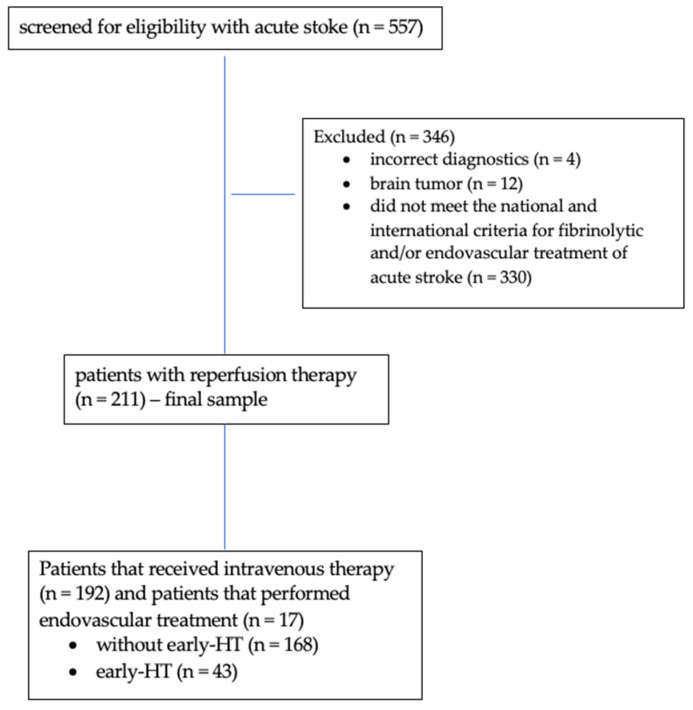
Study flowchart.

As is seen in the study flowchart represented in Figure 2, where a total of 557 patients were screened for eligibility to perform reperfusion therapy, only patients (*n* = 211) that received intravenous thrombolytic therapy (*n* = 192) or endovascular treatment through mechanic thrombectomy (*n* = 17) were included in the final sample of this study. Twelve patients were excluded because the CT examination revealed brain tumors, and four because of incorrect diagnostics. The rest of the 330 patients were excluded because they did not meet the national and international criteria for fibrinolytic and/or endovascular treatment of acute stroke. Among 211 consecutive patients included in the final sample, 43 (20.37%; age: median 70.00 years; 51.2% males) had early HT and 168 (79.63%; age: median 68.00 years; 61.3% males) were without early HT (Figure 2).

### 3.2. Patient Population and Characteristics

Table 1 contains, described in detail, the clinical, biochemical, and demographic features of the patients at admission to reperfusion therapies.

Patients with early HT were older compared with patients without early HT, but no statistically significant difference (*p* = 0.843) was observed between the groups. They also tended to have a higher BMI compared to the other group (without early HT) (*p* = 0.764). Regarding gender, no difference between groups was observed (*p* = 0.231). Although no statistically significant differences were observed between groups in terms of systolic blood pressure (*p* = 0.946) and diastolic blood pressure (*p* = 0.768) in the early-HT group 79.1% of patients (*n* = 34) had hypertension. In the early-HT group, 58.1% of patients (*n* = 25) had T2DM previously, with a statistically significant difference (*p* = 0.007) between the two groups. Moreover, 37.2% of the patients from the early-HT group (*n* = 16) had atrial fibrillation previously, compared with 28.6% (*n* = 48) from the group without early HT (*p* = 0.272). No statistically significant difference was observed between groups for alcohol consumption (*p* = 0.433), smoking (*p* = 0.839), platelets (*p* = 0.279), hemoglobin (*p* = 0.527), creatinine (*p* = 0.098), and INR (*p* = 0.538).

### 3.3. Analysis of Correlations between Stroke Scores and Early HT

The values of ASPECTS at admission and ASPECTS at 24 h are significantly lower in patients with early HT (Mann–Whitney test, *p* = 0.045 and *p* < 0.001, respectively). NIHSS values at admission, at 1 h, 2 h, and 24 h are significantly increased in those with early HT (Mann–Whitney test, *p* < 0.001, *p* = 0.040, *p* = 0.032, and *p* = 0.007, respectively) (Table 2).

### 3.4. Analysis of Correlations between Type of Reperfusion Therapy and Early HT

By applying the chi-square test to analyze the incidence of early HT after the two types of reperfusion therapy (fibrinolytic and endovascular treatment), no statistically significant differences were observed in the study sample between them (*p* = 0.089) (Table 3).

### 3.5. Logistic Regression of Independent Risk Factors Associated with Early HT

In order to identify the independent risk factors for hemorrhagic transformation, we employed a multivariate logistic regression. The odds ratio and 95% confidence interval were calculated and the results are presented in Table 4. The independent factors that increase the risk for hemorrhagic transformation were male gender by 2.7-fold, presence of baseline high blood pressure by 2.4-fold, and high glycemic values by 1.2-fold.

In order to identify the scoring systems that are predictive of the hemorrhagic transformation, a multivariate logistic regression was employed. The odds ratio and 95% confidence interval were calculated and the results are presented in Table 5. Higher values of ASPECTS at 24 h decrease the risk of hemorrhagic transformation by 0.6-fold. Higher values of NIHSS at 24 h increased the risk of hemorrhagic transformation by 1.18-fold.

### 3.6. Analysis of Independent of Stroke Scores between Groups

**Figure 3:**
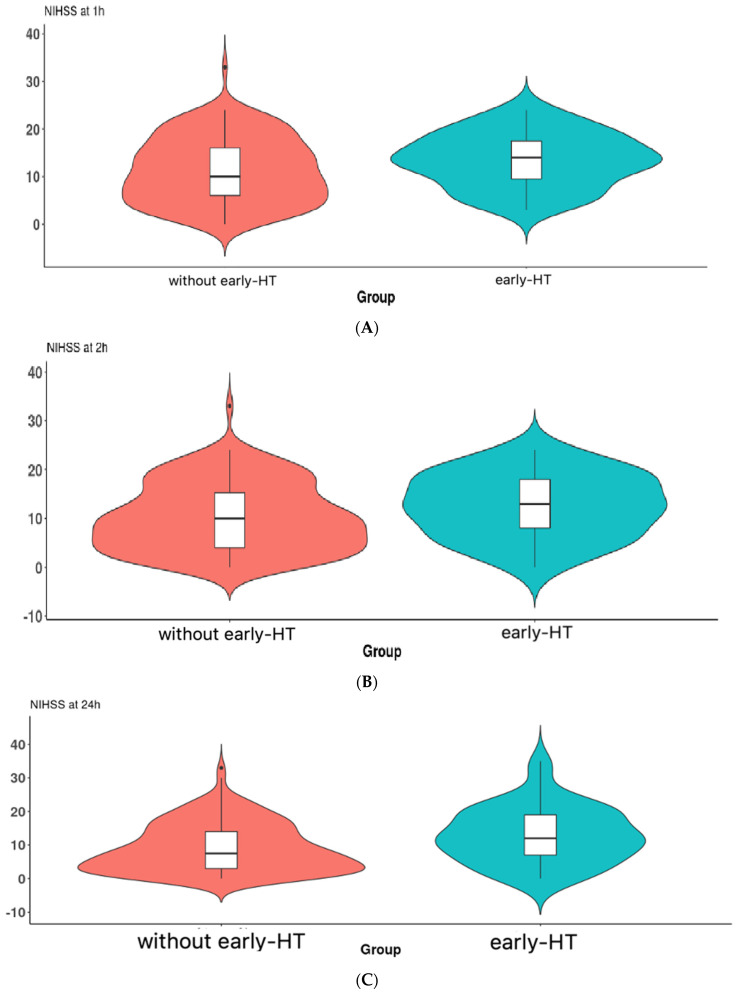
(**A**) Violin plot of the NIHSS at 1 h between the 2 groups. The boxplot inside the violin represents the median and interquartile range. (**B**) Violin plot of the NIHSS at 2 h between the 2 groups. The boxplot inside the violin represents the median and interquartile range. (**C**) Violin plot of the NIHSS at 24 h between the 2 groups. The boxplot inside the violin represents the median and interquartile range.

The median values of NIHSS at 1 h were statistically significantly higher in patients with early HT ((13(6) vs. 14(8), *p* = 0.040, Mann–Whitney test), also at 2 h ((11(5) vs. 14(11), *p* = 0.032, Mann–Whitney test), and at 24 h ((8(10) vs. 13(11), *p* = 0.007, Mann–Whitney test). The frequency distribution of NIHSS values are present in violin plots in Figure 3A–C, respectively.

**Figure 4:**
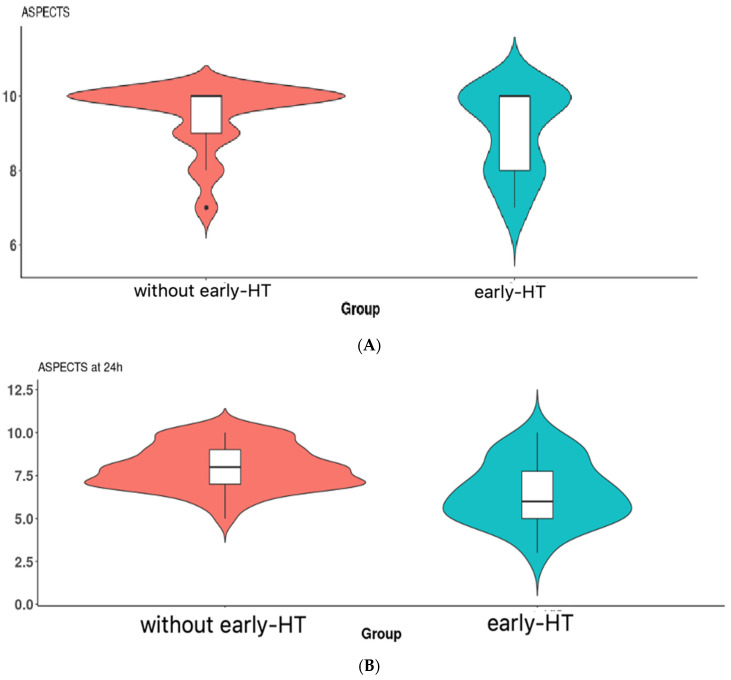
(**A**) Violin plot of the ASPECTS at admission between the 2 groups. The boxplot inside the violin represents the median and interquartile range. (**B**) Violin plot of the ASPECTS at 24 h between the 2 groups. The boxplot inside the violin represents the median and interquartile range.

The median values of ASPECTS at admission were statistically significantly lower in patients with early HT ((9(2) vs. 10(1), *p* = 0.045, Mann–Whitney test), and also at 24 h ((6(3) vs. 8(2), *p* < 0.001, Mann–Whitney test), The frequency distribution of ASPECTS values is present in violin plots in Figure 4A,B.

## 4. Discussion

HT is a major predictor of death and disability in acute ischemic stroke patients undergoing reperfusion therapy. Therefore, this study aimed to identify risk factors, as well as predictors of early HT, as important tools to improve the clinical outcomes of these patients and their quality of life.

Therefore, this study has three major findings. One of our main findings was the identification of certain risk factors as predictors for early HT in patients with AIS treated with reperfusion therapies such as: male gender, baseline high blood pressure and high glycemic values. Another major finding revealed that higher values of NIHSS and lower ASPECTS scores were associated with the increased risk of early HT. Furthermore, from our study results, 20.37% of the patients (*n* = 43; age: median 70.00 years; 51.2% males) had early HT, which represents a relatively high incidence of early HT in this study group compared to other clinical studies.

Several studies have reported different incidences of HT in patients with acute ischemic stroke that performed reperfusion therapies. In a recent multi-centric study conducted by Liu et al. that included 538 patients with ischemic stroke receiving thrombolysis, 17.4% (*n* = 94) were diagnosed with HT on brain computed tomography within 36 h after stroke onset, and half of them (47/94 patients) had symptomatic HT [34]. Another study, on a total of 1207 patients with AIS found that the incidence of HT after 48 h was 14.8% (*n* = 179) and the median time of stroke onset to develop HT was 2 days (1–4 days), between these nine patients (40.9%) were found to have HT on CT scans [35]. A recent study that analyzes HT after mechanical thrombectomy (*n* = 29 patients) vs. mechanical thrombectomy + bolus IV-tPA (*n* = 12 patients) vs. mechanical thrombectomy + full dose IV-tPA (*n* = 7 patients), found that intracranial hemorrhage occurred in 65% of patients who underwent mechanical thrombectomy, in 83% of patients who underwent mechanical thrombectomy + bolus IV-tPA, and in 85% of patients who underwent mechanical thrombectomy + full dose IV-tPA. According to these data, there was no significant difference in terms of the risk of intracranial hemorrhage in patients who received bolus or full dose IV- tPA, but it was observed at a higher rate compared to patients who underwent only mechanical thrombectomy [36]. In our study, when we analyzed the incidence of early HT after IV-tPA therapy (*n* = 35 from 192 patients that performed only fibrinolytic treatment) vs. mechanical thrombectomy (*n* = 6 from 17 patients that performed only endovascular treatment), no statistically significant differences were observed (*p* = 0.089, the chi-square test). Based on these results, we believe that identifying factors that have increased influence on early HT and that could help make a more careful selection of patients for reperfusion therapies is mandatory.

As there is a growing need for the development of predictive models for the selection of patients with low risk for HT regardless of the time frame from symptom onset, several trials have been conducted in order to establish which factors could be predictors of HT for patients with AIS treated with reperfusion therapies. Stroke severity, evaluated with the NIHSS, and the early detection of major infarct changes on brain CT scans, were detected as major predictors of HT in the NINDS trial [37]. A retrospective study by Kidwell et al. also confirmed that higher NIHSS, elevated serum glucose, lower platelet counts, as well as increased time-to-needle, as major HT predictors in AIS. Moreover, this study highlighted the importance of the NIHSS, as higher NIHSS scores predicted larger infarct sizes [38]. NIHSS ≥ 20 was also considered to be a reasonable threshold to predict HT after mechanical thrombectomy by Luo et al. [39].

By analyzing which factors could be predictors of early HT, we found that gender was an important independent early-HT predictor, as males with AIS had a 2.7-fold increased risk for developing early hemorrhagic transformation after reperfusion therapies. Our study finding was also confirmed by a meta-analysis conducted by Lihong Wen et al. in 2020 performed on studies among the Chinese population (*n* = 5597). From their results, risk factors such as male gender, age, diabetes, atrial fibrillation as well as previous strokes were strongly correlated with HT, but also onset-treatment-time (OTT), early infarct changes of CT, and the size of the infarction are associated with the risk of HT [40]. ECASS I trial identified that along with these two factors, increased age could be a predictor of HT, especially for parenchymal hematoma [41].

Among other independent HT risk factors found in our study using multivariate logistic regression, high blood pressure at baseline increased the risk by 2.4-fold and high glycemic values by 1.2-fold. A study by Demchuk et al. discovered that elevated serum glucose is also an independent predictor of HT along with higher NIHSS scores in patients with AIS treated with IV-tPA [42]. Studies have confirmed that high glucose levels are correlated with the presence of HT in acute stroke patients, and also with the disruption of the blood–brain barrier and negative outcomes. While high glucose levels have an initial protective effect on the neuronal cell metabolism at stroke onset, reperfusion via thrombolysis releases the NADPH oxidase from inhibition causing the destruction of the blood–brain barrier due to the generation of superoxide free radicals [43]. This negative effect of high glycemic values has also been observed on cerebral endothelial cells, leading to reduced reperfusion of the microvascular circulation, edema, and thus, hemorrhagic transformation, complicating the final clinical outcome [44]. Current guidelines in AIS reperfusion with IV-tPA demand the reduction of blood pressure below 185/110 mmHg before reperfusion, because of the negative effect of high blood pressure values on the permeability of the blood–brain barrier [45]. In patients with chronic hypertension, cerebral blood vessels have an altered structure, which further aggravates the inflammation caused by superoxide free radicals [46].

We also analyzed the values of the ASPECTS scores at admission and at 24 h, and the results indicated that patients with early HT had significantly lower ASPECTS scores. Furthermore, NIHSS at admission, at 1 h, 2 h, and 24 h was significantly increased in patients with early HT in our study. By using multivariate logistic regression, we identified that higher ASPECTS scores at 24 h decrease the risk of early HT by 0.6 fold. Chang et al. also studied which of the scores used in practice (ASPECTS, DRAGON, SEDAN, and HAT) are the best predictors for a hemorrhagic transformation after IV-tPA, and their results indicated that lower ASPECTS scores were associated with higher rates of HT and that from all studied scores, the predicted value of ASPECTS score is the best [47].

Another recent study on the Chinese population carried out by Shen Z. et al. analyzed predictors of symptomatic intracerebral hemorrhage (sICH) in patients with AIS for patients treated without thrombolysis and found that although the percentage of sICH was very low (0.73%, *n* = 9484), personal history of AF and tumors and NIHSS scores at admission are the main risk factors independently correlated with sICH [48]. In our study, the presence of brain tumors on initial cerebral computer tomography examinations represented an exclusion study criteria, and regarding the history of AF, no statistically significant differences were observed between the two groups (*p* = 0.272) (37.2% patients from the early-HT group vs. 28.6% from the group without early HT).

While thrombolytic agents are the best treatment option in AIS, due to their important side effects such as HT, their association with other methods that wish to monitor and/or improve cerebral reperfusion are also analyzed in the literature. Today, ultrasound technology is developing rapidly and is involved in a growing number of fields [49,50,51,52,53]. The application of ultrasound has extended from traditional diagnosis to treatment and has achieved remarkable results. As the number of patients with brain disease increased, cranial ultrasound techniques advanced from simple morphological imaging to complex functional recovery and treatment [52]. In a meta-analysis by Tsivgoulis et al., transcranial Doppler ultrasound was studied as an effective tool for the improvement of the rtPA effect in AIS. The principle of this technique consists of exposing clots to low- or high-frequency ultrasound, thus enhancing the thrombolytic effect via mechanical force. Although more effective and with promising results as a rtPA enhancer, low-frequency ultrasound was correlated with significantly more cases of intracerebral bleeding [54]. In a study conducted by Eggers et al., on the application of ultrasound thrombolysis to treat acute ischemic stroke, in patients with contraindication for rtPA therapy, where the treatment group underwent ultrasound thrombolysis for over 1 h vs. a control group, the rates of vascular recanalization and neurological improvement were significantly higher in the treatment group than in the control group after 4 days. These results demonstrated that ultrasound thrombolysis is an effective treatment and provides a new treatment option for stroke patients who are not eligible for rtPA-based thrombolytic therapy [55].

Although the ischemic penumbra could be saved in the first 8–12 h, the time limit for rtPA treatment is 4.5 h due to the increase in the blood–brain barrier defect over time, also increasing the risk for HT and especially for parenchymal hematoma. In order for more patients with AIS to benefit from reperfusion therapies, studies today are aimed at extending the time window for rTPA. In order for this to be possible in the future, HT predictors should be analyzed properly in all patients. A review published by Zhang et al. in 2021 analyzed HT predictors and concluded that HT depends on many factors such as infarction localization and size, poor collateral vascularization, early ischemic signs on CT as well as the presence of hyperglycemia, atrial fibrillation, low total cholesterol and LDL-c values, low platelet count and high globulin values [56].

Therefore, early recognition and preliminary assessment of HT with new risk factors such as fibrinogen-to-albumin ratio (FAR) are essential to distinguish patients at risk and to develop new therapies for this complication [57].

*The present study has several limitations.* Due to the design of the retrospective study, only the data collected from electronic and paper medical records could be analyzed for association with hemorrhagic transformation in patients with acute ischemic stroke. The study population was somewhat homogeneous and included consecutive patients treated only with one type of thrombolytic agent (with intravenous rtPA) or with mechanical thrombectomy, and with no statistically significant difference regarding age. Further multi-centric human studies are needed to confirm the effects and investigate the mechanisms of these potential predictors and also to analyze the influence of major risk factors on early HT, in order to develop new methods to prevent and/or reduce HT in AIS. In addition, there are many important aspects regarding the hemorrhagic transformation of AIS in humans that could not be reproduced in animal stroke models [58], patients who often have multiple risk factors and comorbid diseases that are associated with HT cannot be usually modeled in animals [13].

## 5. Conclusions

Recognition of risk factors for early HT may reduce the incidence and severity of complications related to reperfusion therapies in AIS. The present study identified and reconfirmed certain risk factors as predictors for early HT in patients with AIS treated with reperfusion therapies such as male gender, baseline high blood pressure, and high glycemic values. Moreover, patients with higher values of NIHSS and lower ASPECTS scores were associated with an increased risk of early HT.

It is of critical importance to identify predictors of HT in patients with AIS receiving reperfusion therapies in order to minimize the risk of negative outcomes. Predictive models could be used in the future for the selection of patients with low risk of complications time-of-onset independent. The identification of HT predictors is important for the development of future therapies in AIS use for patients that are not eligible for or have a high risk of complications with current reperfusion therapies (thrombolysis and mechanical thrombectomy).

## Figures and Tables

**Table 1:** Characteristics of all patients (*n* = 211).

Variables	Without Early HT *n* = 168	Early HT *n* = 43	*p* Value
**Age, years**	68 (15)	70 (15)	0.843 ^M-W^
**Male sex, *n* (%)**	103 (61.3%)	22 (51.2%)	0.227 ^Chi2^
**BMI, kg/m^2^**	26.1 (5)	27.5 (5.2)	0.764 ^M-W^
**SBP, mmHg**	153.3 ± 22.83	152.4 ± 19.64	0.946 ^unpT^
**DBP, mmHg**	80 (20)	80 (20)	0.767 ^M-W^
**Hypertension, *n* (%)**	127 (75.6%)	34 (79.1%)	0.633 ^Chi2^
**T2DM, *n* (%)**	79 (47.0%)	25 (58.1%)	0.193 ^Chi2^
**Dyslipidemia, *n* (%)**	66 (39.3%)	17 (39.5%)	0.976 ^Chi2^
**AF, *n* (%)**	48 (28.6%)	16 (37.2%)	0.272 ^Chi2^
**Smoking, *n* (%)**	52 (31.0%)	14 (32.6%)	0.839 ^Chi2^
**Alcohol, *n* (%)**	52 (31.0%)	16 (37.2%)	0.433 ^Chi2^
**Platelets count (×10^9^ /L)**	213 (81)	229 (87)	0.279 ^M-W^
**Hemoglobin (g/dL)**	14 (2)	14 (2)	0.527 ^M-W^
**Creatinine (mg/dL)**	1.0 ± 0.25	1 (0)	0.098 ^M-W^
**TC, mg/dL**	183.5 ± 48.84	185.9 ± 39.60	0.699 ^unpT^
**Blood glucose (mg/dL)**	131.01 ± 46.04	154.35 ± 63.57	0.007 ^unpT^
**INR**	1.05 ± 0.15	1.04 ± 0.10	0.538 ^unpT^

SBP, systolic blood pressure; DBP, diastolic blood pressure; BMI, Body mass index; AF, atrial fibrillation; T2DM, type 2 diabetes mellitus; TC, total cholesterol; INR, international-normalized ratio; Values were expressed as mean ± standard deviation (SD). ^unpT^—unpaired *t*-Test for continue variable with Gaussian distribution—data represented by mean ± std. dev.; ^M-W^—Mann–Whitney U Test for continuing variable without Gaussian distribution—data represented by median (interquartile range); ^Chi2^—CHI^2^ Test for nominal variables—data represented by numbers (%).

**Table 2:** Stroke Score of Hemorrhagic Transformation for all patients (*n* = 211).

Variables	Without Early HT *n* = 168	Early HT *n* = 43	*p* Value
**ASPECTS at admission**	10 (1)	9 (2)	0.045 ^M-W^
**ASPECTS at 24 h**	8 (2)	6 (3)	<0.001 ^M-W^
**NIHSS at admission**	13 (9)	14 (6)	<0.001 ^M-W^
**NIHSS at 1 h**	13 (6)	14 (8)	0.040 ^M-W^
**NIHSS at 2 h**	11 (5)	14 (11)	0.032 ^M-W^
**NIHSS at 24 h**	8 (10)	13 (11)	0.007 ^M-W^

^M-W^—Mann–Whitney U Test for continuing variable without Gaussian distribution—data represented by median (interquartile range).

**Table 3:** Type of reperfusion therapy for all patients (*n* = 211).

Variables	Without Early HT *n* = 168	Early HT *n* = 43	*p* Value
**I** **ntravenous thrombolytic treatment (*n* = 192)**	157 (154.33)	35 (37.67)	0.089
**Endovascular treatment (*n* = 17)**	11 (13.67)	6 (3.33)	

**Table 4:** Logistic Regression (using Enter method) considering early HT as a dependent variable and risk factors as independent variables.

Variable	*p* Values	Adjusted OR (95% CI)
**Male sex (yes)**	0.035	2.736 (1.986–7.591)
**Age, years**	0.294	1.022 (0.981–1.064)
**BMI, kg /m^2^**	0.911	1.006 (0.909–1.113)
**Hypertension (yes)**	0.037	2.455 (1.481–4.403)
**T2DM (yes)**	0.212	0.585 (0.252–1.357)
**Dyslipidemia (yes)**	0.460	1.387 (0.582–3.310)
**AF (yes)**	0.644	0.816 (0.346–1.929)
**Smoking (yes)**	0.629	1.336 (0.412–4.331)
**alcohol (yes)**	0.268	0.525 (0.168–1.641)
**SBP, mmHg**	0.478	0.993 (0.972–1.013)
**DBP, mmHg**	0.513	1.010 (0.980–1.042)
**Platelets count (×10^9^/L)**	0.327	1.003 (0.997–1.008)
**Hemoglobin (g/dL)**	0.195	1.188 (0.916–1.541)
**Blood glucose (mg/dL)**	0.042	1.207 (1.100–1.915)
**TC, mg/dL**	0.868	1.001 (0.992–1.010)

**Table 5:** Logistic Regression (using Enter method) considering early HT as a dependent variable and scores as independent variables.

Scores	*p* Value	Adjusted OR (95% CI)
**ASPECTS at admission**	0.906	0.969 (0.575–1.632)
**ASPECTS at 24 h**	0.016	0.601 (0.396–0.911)
**NIHSS at admission**	0.945	0.090 (0.953–1.154)
**NIHSS at 1 h**	0.927	1.007 (0.877–1.156)
**NIHSS at 2 h**	0.936	0.994 (0.859–1.150)
**NIHSS at 24 h**	0.027	1.186 (1.109–2.169)

## Data Availability

The data that support the findings of this study are available from the corresponding author upon reasonable request.
